# CD73 facilitates EMT progression and promotes lung metastases in triple-negative breast cancer

**DOI:** 10.1038/s41598-021-85379-z

**Published:** 2021-03-16

**Authors:** Nataliia Petruk, Sanni Tuominen, Malin Åkerfelt, Jesse Mattsson, Jouko Sandholm, Matthias Nees, Gennady G. Yegutkin, Arja Jukkola, Johanna Tuomela, Katri S. Selander

**Affiliations:** 1 Institute of Biomedicine, University of Turku, Turku, Finland; 2 Western Cancer Centre FICAN West, Turku, Finland; 3 Preclinical Imaging Laboratory, Turku PET Centre, University of Turku, Turku, Finland; 4 Faculty of Science and Engineering, Cell Biology, Åbo Akademi University, Turku, Finland; 5 Turku Bioscience Centre, University of Turku and Åbo Akademi University, Turku, Finland; 6 Department of Biochemistry and Molecular Biology, Medical University in Lublin, Lublin, Poland; 7 MediCity Research Laboratory, University of Turku, Turku, Finland; 8 Department of Oncology, Tampere University Hospital, Tays Cancer Center, Tampere, Finland; 9 Turku, Finland; 10 Department of Oncology, Oulu University Hospital, Oulu, Finland

**Keywords:** Breast cancer, Epithelial-mesenchymal transition, Cell migration

## Abstract

CD73 is a cell surface ecto-5′-nucleotidase, which converts extracellular adenosine monophosphate to adenosine. High tumor CD73 expression is associated with poor outcome among triple-negative breast cancer (TNBC) patients. Here we investigated the mechanisms by which CD73 might contribute to TNBC progression. This was done by inhibiting CD73 with adenosine 5′-(α, β-methylene) diphosphate (APCP) in MDA-MB-231 or 4T1 TNBC cells or through shRNA-silencing (sh-CD73). Effects of such inhibition on cell behavior was then studied in normoxia and hypoxia in vitro and in an orthotopic mouse model in vivo. CD73 inhibition, through shRNA or APCP significantly decreased cellular viability and migration in normoxia. Inhibition of CD73 also resulted in suppression of hypoxia-induced increase in viability and prevented cell protrusion elongation in both normoxia and hypoxia in cancer cells. Sh-CD73 4T1 cells formed significantly smaller and less invasive 3D organoids in vitro, and significantly smaller orthotopic tumors and less lung metastases than control shRNA cells in vivo. CD73 suppression increased E-cadherin and decreased vimentin expression in vitro and in vivo, proposing maintenance of a more epithelial phenotype. In conclusion, our results suggest that CD73 may promote early steps of tumor progression, possibly through facilitating epithelial–mesenchymal transition.

## Introduction

Triple-negative breast cancer (TNBC) is characterized by the lack of estrogen, progesterone receptor expression, HER2 amplification and represents ~ 15–20% of all breast cancers. Despite recent advances with targeted cancer treatments, TNBC patients continue to have limited treatment options, with chemotherapy, surgery and radiation therapy remaining as the standard of care^1–4^.

CD73 is a cell surface ecto-5′-nucleotidase, which converts extracellular adenosine monophosphate (AMP) to adenosine and inorganic phosphate^5,6^. Adenosine is an anti-inflammatory agent, which prevents excess inflammatory reactions and has been shown to be a potential target for autoimmune diseases^7,8^. CD73 is expressed in various cancers, including breast cancer^9–12^. Moreover, several studies have demonstrated that CD73 has prognostic value in TNBC^13–15^. No such correlation was detected among HER2+ or luminal breast cancer subtypes^15,16^. CD73-associated poor outcome in TNBC may stem from immune evasion, as adenosine may protect cancer cells from adaptive anti-tumor immune responses^17–19^. Recent studies have, however, also demonstrated, that CD73 promotes cell migration, invasion and, possibly due to its immunosuppressive capability, chemotherapy resistance^13,14,20^.

Tumor microenvironment is typically hypoxic, which promotes tumor survival by enhancing angiogenesis and metastasis, and reducing apoptosis^21–23^. Hypoxia may also regulate epithelial–mesenchymal transition (EMT), which is important in tumor progression^24^. In cancer, hypoxia induces CD73 expression through hypoxia-inducible factor-1α (HIF-1α) activation and their expressions positively correlate in clinical cancer samples^14,25^. Recent studies associated CD73 expression with EMT regulation in cancer^9, 26,27^. The aim of this study was to further investigate the mechanisms how CD73 may contribute to tumor progression.

## Results

### CD73 suppression inhibits cell viability and proliferation in normoxia

**Figure 1:**
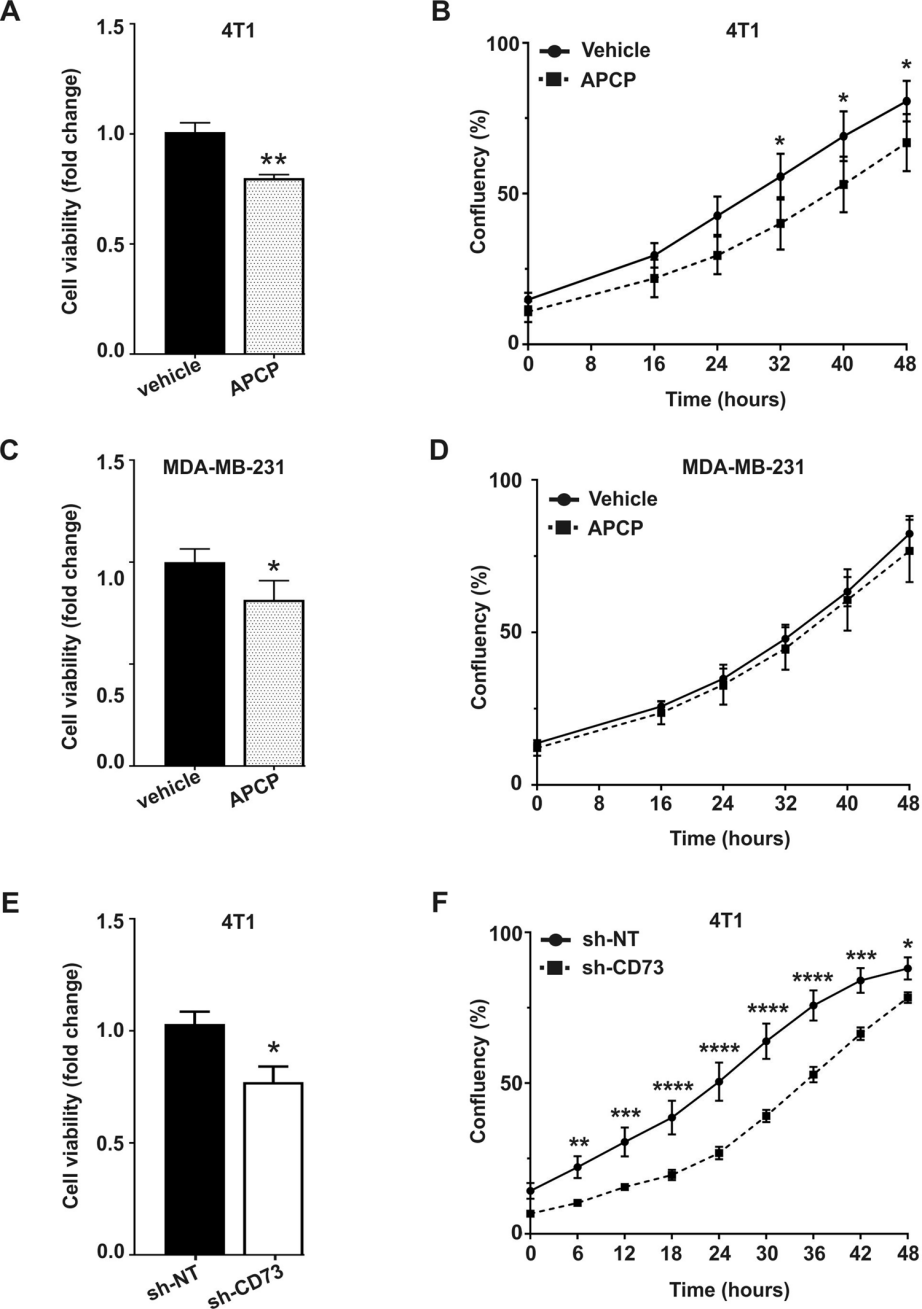
CD73 suppression inhibits cell viability and proliferation in normoxia. Cell viability was measured by WST-8 assay. Cell proliferation was assessed with confluence analysis. Viability (**A**) and proliferation (**B**) of APCP-treated 4T1 cells in normoxia. Viability (**C**) and proliferation (**D**) of APCP-treated MDA-MB-231 cells in normoxia. Viability (**E**) and proliferation (**F**) of sh-NT (control) and sh-CD73 4T1 cells in normoxia. The bars represent fold-change in viability vehicle-treatment (**A**,**C**), or vs. sh-NT cells (**E**). Cell proliferation rates are expressed as percentages of confluency (**B**,**D**,**F**). The cell confluency was analyzed using IncuCyte 2018B software (Essen Bioscience). The results are expressed as mean ± SD, n = 3. *P < 0.05, **P < 0.01 and ***P < 0.001 are considered to be statistically significant compared to representative control cells, by two-tailed Student’s t-test.

To determine the role of CD73 enzymatic activity and expression in TNBC, two approaches to suppress CD73 were applied. In the first approach, CD73 activity was inhibited with the inhibitor APCP (Fig. S1A). Additionally, CD73 activity was significantly decreased in 4T1 sh-CD73 compared to sh-NT cells (Fig. S1B). CD73 expression was suppressed in 4T1 cells by lentiviral shRNA constructs (Fig. S1C–F). APCP significantly decreased 4T1 cell viability (p = 0.0063) and proliferation (p = 0.0477) in normoxia (Fig. 1A,B). APCP also significantly decreased viability (p = 0.0122) but not proliferation (p = 0.6978) of MDA-MB-321 cells in normoxia (Fig. 1C,D). Similarly, suppression of CD73 expression significantly decreased viability (p = 0.0286) and proliferation (p = 0.0196) of 4T1 cells in normoxia (Fig. 1E,F).

### CD73 inhibition dampens hypoxia-induced increase in cell viability

**Figure 2:**
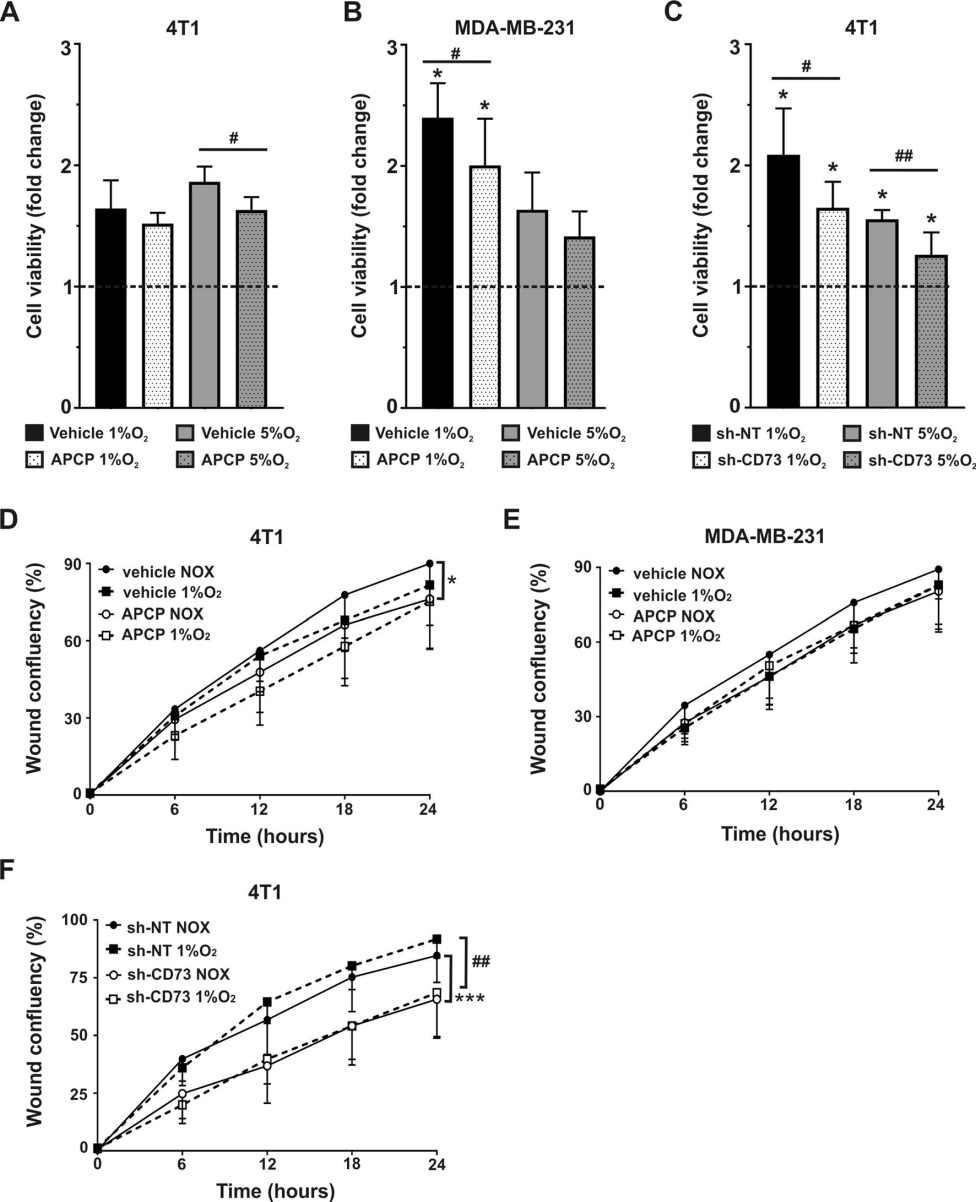
CD73 suppression inhibits cell viability and migration in hypoxia. (**A**) Viability of APCP- or vehicle-treated 4T1 and (**B**) APCP- or vehicle-treated MDA-MB-231 cells, (**C**) sh-NT and sh-CD73 4T1 cells, were measured by WST-8 assays. The bars in (**A**–**C**) represent fold-change in viability, as compared with normoxia (set to 1 and indicated with the dotted line). The bars represent mean ± SD, n = 3. *P < 0.05 vs the same cells in normoxia. ^#^P < 0.05, ^##^P < 0.01; sh-CD73 cells vs. sh-NT cells, or APCP vs. vehicle, by one-way ANOVA with a Dunnett post-test. Scratch wound assay with (**D**) APCP- or vehicle-treated 4T1, (**E**) APCP-or vehicle-treated MDA-MB-231 cells, and (**F**) sh-NT and sh-CD73 4T1 cells, in normoxia or 1% O_2_ for 24 h. Wound confluences were analyzed using IncuCyte 2018B software (Essen Bioscience). The results are expressed as mean ± SD, n = 3. *P < 0.05; ***P < 0.001; sh-CD73 or APCP-treated 4T1 cells vs representative controls in normoxia; or ^##^P < 0.01 in hypoxia, by one-way ANOVA with a Dunnett post-test.

As tumors typically have hypoxic areas with varying oxygen levels^22^, we further investigated the effects of 1% and 5% hypoxia on CD73 expression, viability and migration in 4T1 and MDA-MB-231 cells. As expected, hypoxic conditions with 1% O_2_ were confirmed to increase HIF-1α and specificity protein 1 (SP1) mRNA expression levels (Fig. S2A,B). Furthermore, as expected based on a previous publication, hypoxia upregulated CD73 expression in both parental 4T1 and MDA-MB-231 cells (Fig. S2C,D)^28^. Hypoxia increased the viability of parental 4T1 and MDA-MB-231 as expected. Both 1% and 5% hypoxia also significantly increased the viability of both 4T1 sh-NT (p = 0.0313) and sh-CD73 cells (p = 0.0313), as compared with their viability in normoxia (Fig. 2C). However, APCP significantly inhibited 5% hypoxia-induced increase in viability of 4T1 cells, when compared with that of untreated cells (p = 0.0159) (Fig. 2A). A similar effect was also seen in APCP-treated parental MDA-MB-231 cells in 1% hypoxia (p = 0.0411 vs. untreated cells) (Fig. 2B). Similarly, the hypoxia-induced increase in viability of 4T1 sh-CD73 cells in response to 1% or 5% O_2_, was significantly smaller than that detected in sh-NT cells (p = 0.026 and p = 0.043, respectively) (Fig. 2C). Taken together, our data shows that hypoxia-induced increase in cell viability is dependent on CD73 expression or enzymatic activity.

### CD73 suppression inhibits TNBC cell migration in both normoxia and hypoxia

Migration was assessed with wound healing assays in both normoxia and hypoxia. Inhibition of CD73 activity through APCP diminished 4T1 migration in normoxia (Fig. 2D, APCP vs. vehicle, p = 0.0112). APCP did not inhibit MDA-MB-231 migration (Fig. 2E). However, suppression of CD73 expression significantly reduced 4T1 migration, as compared with sh-NT cells, both in normoxia and hypoxia (p = 0.0009) (Fig. 2F). 1% O_2_ had no effect on parental 4T1 or MDA-MB-231 cell migration (Fig. 2D,E). Furthermore, hypoxia effects on migration were comparable between 1% and 5% O_2_ in these cells (Fig. S3A,B). Taken together, inhibition of CD73 expression or activity inhibits 4T1 cell migration in both normoxia and hypoxia.

### CD73 suppression inhibits organoid formation in a 3D-model

**Figure 3:**
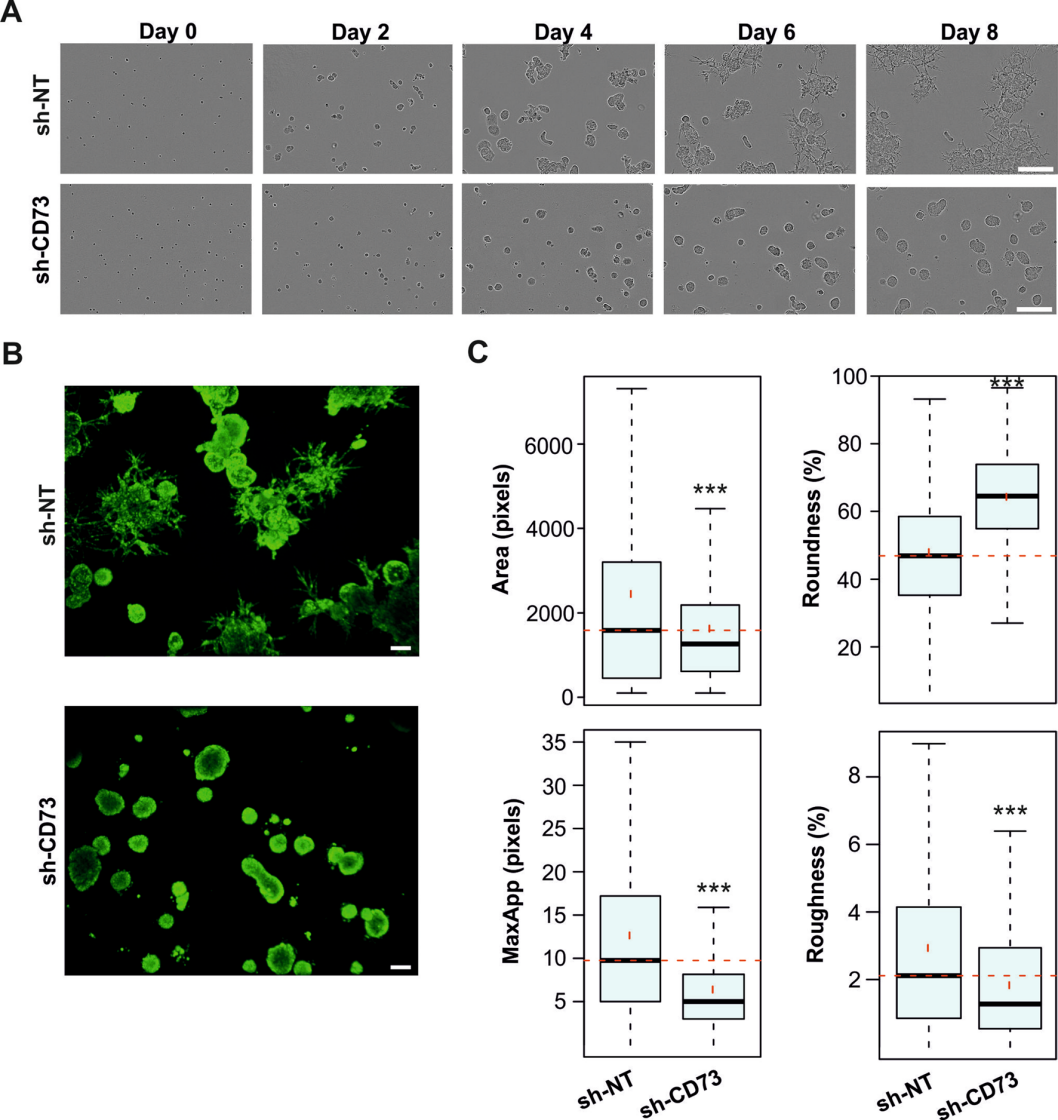
CD73 suppression prevents TNBC organoid invasion. (**A**) Sh-NT and sh-CD73 4T1 cells were grown in 3D culture. Representative images from days 0 to 8 are shown. Scale bar: 300 μm. (**B**) Confocal spinning disk microscopy images of sh-NT and sh-CD73 3D culture after 6 days. Calcein AM was used to detect living cells (green). Scale bar: 100 μm. (**C**) Box and whisker plots of selected parameters. Spheroid growth is indicated by the Area of the organoids. Roundness serves as a measure for the loss of the round organoid phenotype, and is associated with invasive properties. Roughness of the segmented structures and the maximal length of cellular protrusions emerging from the core structure (MaxApp) are quantitative measures for local invasion and cell motility. ***P < 0.001; sh-CD73 vs sh-NT spheroid formation, n = 1038 sh-NT organoids analyzed, n = 1080 sh-CD73 organoids analyzed; mean ± SD, by a Bonferroni-corrected post-test from T-tests. Data represents two independent experiments.

To assess whether CD73 suppression affects tumor growth and morphology, an organotypic 3D cell culture model was used. Different ratios of Matrigel mixed with collagen I were used to provide ECM conditions that promote both the differentiation, polarization and local invasion of 4T1 cells. Based on these experiments, we chose to proceed with a Matrigel to collagen I ratio of 8:2 (Fig. S4A,B). Organoids developed from single sh-NT cells started to spontaneously invade on or around day 6, followed by evident invasion on day 8 of 3D-culture. The sh-CD73 organoids remained, however, well-differentiated, and did not develop invasive properties, as compared with sh-NT organoids (Fig. 3A). Invasion was not observed even on day 13 in sh-CD73 organoids (Fig. S4C). In order to quantify critical morphologic parameters, organoids were stained with Calcein AM (Fig. 3B). Sh-CD73 cells formed significantly smaller organoids (Area) and exhibited less invasive features (Roundness). The sh-CD73 organoids had also significantly smaller number and length (MaxApp) of invasive multicellular structures as well as the number of small filopodia-like cellular extensions (Roughness), as compared with sh-NT organoids (p < 0.0001) (Fig. 3C). Similar effects were seen in hypoxia (Fig. S5). These results further suggest that CD73 may regulate tumor cell motility and invasion during early steps of cancer progression.

### CD73 facilitates EMT progression in TNBC cells

**Figure 4:**
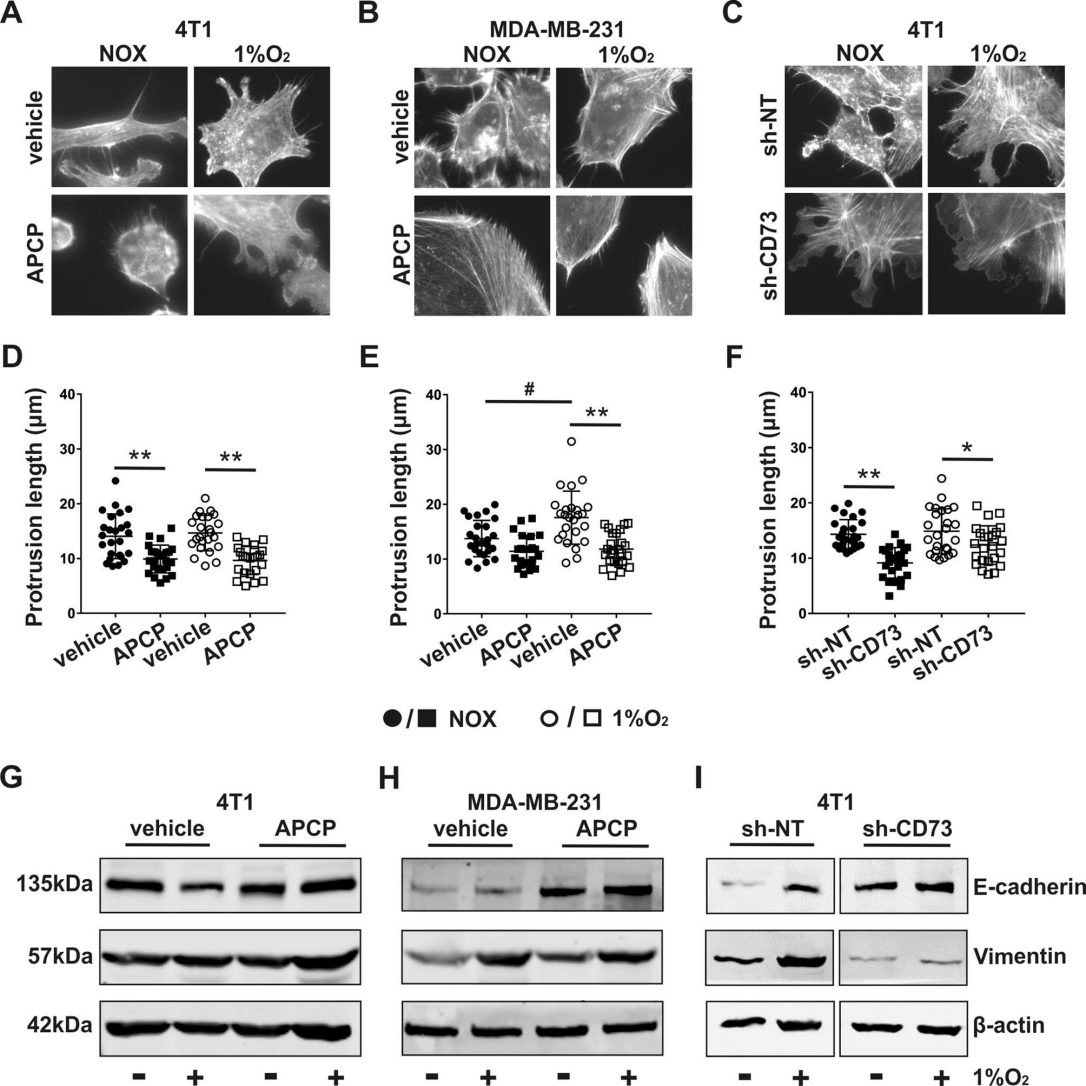
Suppression of CD73 inhibits EMT. Representative images of F-actin immunostaining from (**A**) APCP-treated 4T1 cells, (**B**) MDA-MB-231 cells and (**C**) sh-NT and sh-CD73 4T1 cells in normoxia and 1% O_2_. Quantification of protrusion lengths of (**D**) APCP-treated 4T1 cells and (**E**) MDA-MB-231 cells and (**F**) sh-NT and sh-CD73 4T1 cells, in normoxia and 1% O_2_. The results are expressed as mean ± SD from 3 independent experiments, n = 25 cells analyzed in each group. E-cadherin and vimentin protein expressions in (**G**) APCP- or vehicle-treated 4T1 cells, (**H**) APCP- or vehicle-treated MDA-MB-231 cells and (**I**) sh-NT and sh-CD73 4T1 cells. The blots were taken from different parts of the same gel. The results are expressed as mean ± SD, n = 3. *P < 0.05 and **P < 0.001 vs representative controls, by a two-tailed Student’s t-test; ^#^P < 0.05; vehicle- vs. APCP-treated cells under 1% O_2_.

We next investigated whether the differences between sh-NT and sh-CD73 cells detected in the migration assays and in the 3D organoid model are associated with cell protrusion lengths. APCP treatment significantly shortened protrusions in parental 4T1 cells both in normoxia (p = 0.0090) and in 1% O_2_ (p = 0.0062), as compared with vehicle treatment (Fig. 4A,D). In parental MDA-MB-231 cells, hypoxia increased protrusion lengths (p = 0.017), and this was significantly inhibited by APCP in 1% O_2_ (p = 0.0017) but not in normoxia (p = 0.124) (Fig. 4B,E). Similarly, the cell protrusions were significantly shorter in the 4T1 sh-CD73 cells compared with the sh-NT cells in normoxia (p = 0.0050) and also in 1% O_2_ (p = 0.0444) (Fig. 4C,F).

We then studied whether the changes in protrusion lengths correlate with changes that occur in EMT development. APCP-treated parental 4T1 or MDA-MB-231 and sh-CD73 4T1 cells exhibited increased E-cadherin expression in normoxia, as compared with the corresponding control cells. Similarly, E-cadherin expression levels were increased in 1% O_2_ in APCP-treated parental 4T1 and MDA-MB-231 cells, as well as in sh-CD73 4T1 cells, when compared with the corresponding control cells in 1% hypoxia, but the increase was not statistically significant. Hypoxia increased vimentin levels in all cell lines compared with their corresponding controls in normoxia. Vimentin expression decreased in the sh-CD73 cells, compared with sh-NT cells, both in normoxia and hypoxia, but the decrease was not statistically significant. A similar trend was detected in APCP-treated parental MDA-MB-231 cells, when compared with the corresponding controls in hypoxia. In general, however, the effects of APCP on vimentin expression were not as clear as with the sh-RNA approach (Figs. 4G–I and S6). Our findings thus suggest that CD73 suppression results in inhibition of cell protrusion elongation, increased E-cadherin expression and decreased vimentin expression, especially in normoxia. Taken together, these results suggest that CD73 expression may promote EMT in vitro.

### CD73 promotes tumor growth and lung metastasis

**Figure 5:**
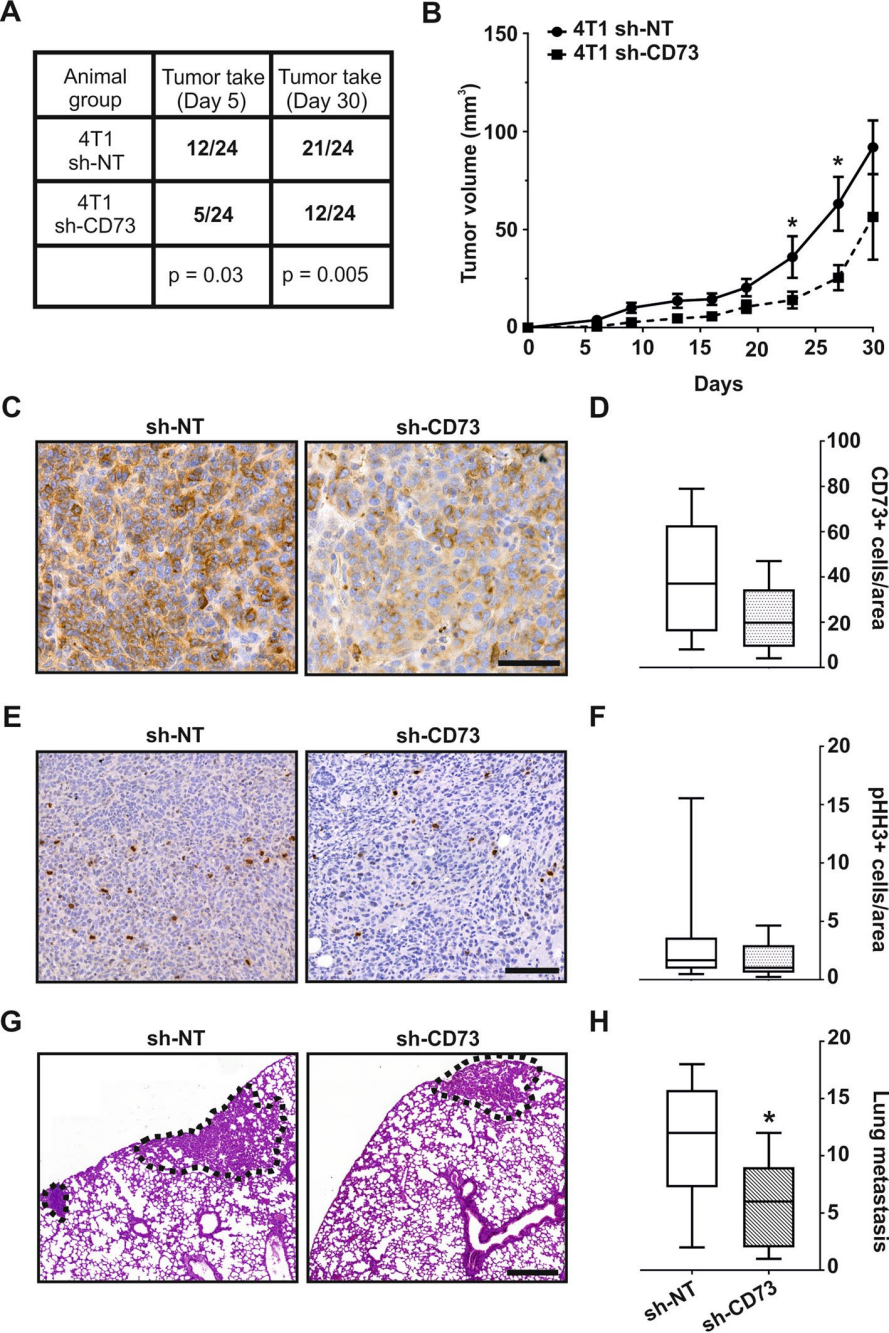
CD73 suppression inhibits tumor take, growth and lung metastases. (**A**) sh-NT and sh-CD73 4T1 cell tumor take rates. The numbers indicate formed tumors per 24 tumor inoculations. P-values were analyzed by Chi-Square test. (**B**) sh-NT and sh-CD73 tumor growth demonstrated as a function of time. (**C**) Representative images of CD73-positive cells in sh-NT and sh-CD73 tumors. Scale bar 100 µm. (**D**) Percentage of CD73-positive cells from 4T1 sh-NT and sh-CD73 tumors. (**E**) Representative images of pHH3-positive cells as an indicator of proliferation in sh-NT and sh-CD73 tumors. Scale bar 200 µm. (**F**) Percentage of pHH3-positive cells from 4T1 sh-NT and sh-CD73 tumors. Immunohistochemical staining expressions were quantified by QuPath. (**G**) Representative H & E stainings from the lungs of tumor bearing mice, metastases are indicated with dotted line. Scale bar 500 µm. (**H**) Numbers of lung metastases from sh-NT and sh-CD73 groups. Data is expressed as mean ± SEM, by a two-tailed Student’s t-test. *P < 0.05; sh-CD73 vs. sh-NT tumors.

To study the role of CD73 in vivo, we used an immunocompetent TNBC model, where 4T1 sh-NT or sh-CD73 cells were inoculated orthotopically into the mammary fat pads of BALB/c mice. Thirty days after tumor cell inoculation, tumor take rate was 88% in the sh-NT group. Surprisingly, tumor take rate in the sh-CD73 group was significantly lower, and seen in only half of the mice (p = 0.005) (Fig. 5A). In agreement with the increased viability and organoid formation in vitro, sh-NT tumors exhibited significantly faster growth (p = 0.012) in vivo compared to tumors with suppressed CD73 expression (Fig. 5B–D). In line with the increased tumor sizes, the number of mitotic cells, as indicated by pHH3-expressing cells, was higher in the sh-NT group than in the sh-CD73 group, but the difference did not reach statistical significance (p = 0.876) (Fig. 5E,F). Decreased migration, as seen in sh-CD73 cells in vitro, was consistent with the significantly decreased number of lung metastases detected in the sh-CD73 tumor-bearing mice (p = 0.0471) (Fig. 5G,H). The number of liver metastases was also lower, but the difference did not reach statistical significance (Fig. S7).

### CD73 suppression inhibits EMT in vivo

**Figure 6:**
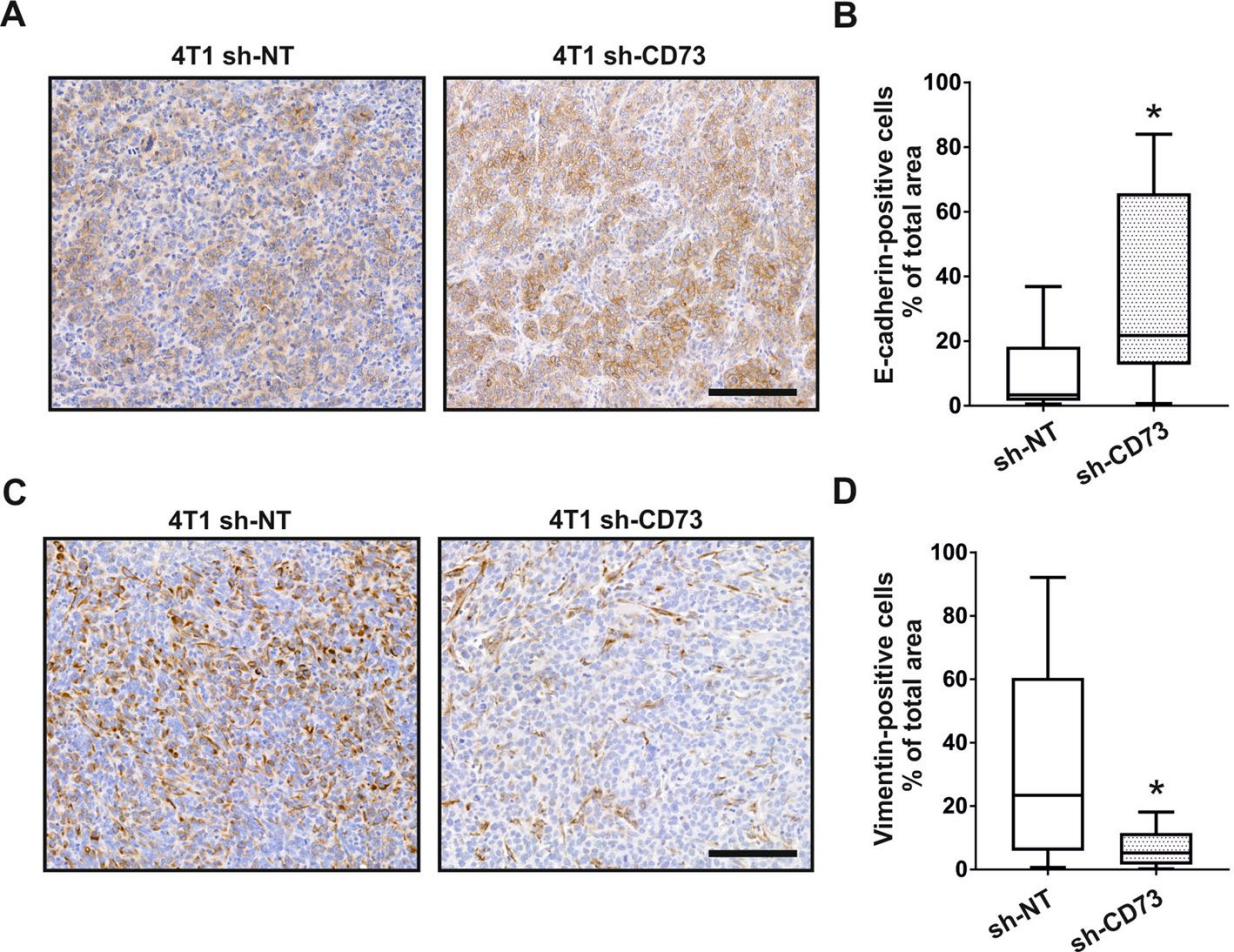
CD73 suppression enhances E-cadherin expression and inhibits vimentin expression in vivo. (**A**) Representative images of E-cadherin-positive cells in sh-NT and sh-CD73 tumors. Scale bar 200 µm. (**B**) Percentage of E-cadherin-positive cells from 4T1 sh-NT and sh-CD73 tumors. (**C**) Representative images of vimentin-positive cells in sh-NT and sh-CD73 tumors. Scale bar 200 µm. (**D**) Percentage of vimentin-positive cells from 4T1 sh-NT and sh-CD73 tumors. For each staining, the expression was quantified using  QuPath. Data is expressed as mean ± SEM, *P < 0.05; sh-CD73 vs. sh-NT tumors, by a two-tailed Student’s t-test.

To further investigate whether suppressed metastasis of sh-CD73 cells is associated with EMT also in vivo, we studied E-cadherin and vimentin expression in the tumors with immunohistochemistry. Tumors with suppressed CD73 expression demonstrated significantly increased E-cadherin expression, as compared with the sh-NT group (p = 0.0328) (Fig. 6A,B). Sh-CD73 tumors also exhibited significantly reduced vimentin expression (p = 0.0159) (Fig. 6C,D). We further investigated whether CD73 expression correlates with EMT in the patient specimens. To test this, we compared the expression of mRNAs encoding CD73 with E-cadherin and vimentin expression in 1904 breast cancer specimens from the METABRIC study (Molecular Taxonomy of Breast Cancer International Consortium) database available in cBioPortal^29–31^. In agreement with our pre-clinical data, there was a statistically significant, direct correlation between CD73 and vimentin mRNA, but not with CD73 and E-cadherin mRNA (Fig. S8). Taken together, our results indicate that CD73 suppression inhibits tumor growth and metastasis, which may be associated with inhibition of EMT in vivo.

## Discussion

TNBC is a molecularly heterogeneous disease, consisting of various subtypes, which demonstrate differences in molecular profiles, immune infiltrates and sensitivity to chemotherapy^2,32–34^.These findings have led to several, ongoing clinical trials targeting subtype specific molecular TNBC pathways^34^. Nevertheless, surgery, chemotherapy and radiation remain the standard of care in early-stage unselected TNBC patients and clearly, there is an unmet need to improve treatment for those TNBC patients that fare the poorest^31^.

Low tumor CD73 expression in cell is associated with prolonged disease-free survival compared to high CD73 expression among TNBC patients^15^. We studied here further the possible cellular mechanisms through which CD73 might promote TNBC, beside its effect on immunosuppression. In agreement with previous publications, our data shows that suppression of CD73 expression and activity inhibits TNBC viability, proliferation and migration in normoxia in vitro. Hypoxia typically develops once the tumors grow beyond 1 cm^35,36^. Furthermore, hypoxia is also one of the factors that accelerate the production of ATP, which is rapidly converted to adenosine resulting in an anti-inflammatory response^7,8,37^. Our results demonstrated, that the inhibitory effects of the lack of CD73 on cell viability were hypoxia-dependent. The effects on migration were, however, independent of hypoxia. We also compared tumor forming capacities of sh-NT and sh-CD73 cells in an organotypic 3D model. This model promotes organotypic acinar structures or organoids, with physiologically relevant cell–cell and cell–matrix interactions, epithelial polarization and differentiation, thus recapitulating tumor histology in vivo^38,39^. With this model, we demonstrated that invasion was blocked in sh-CD73 organoids, suggesting that CD73 may have a role in early tumor progression. Furthermore, we found that CD73 suppression inhibits elongation of cellular protrusions in normoxia and in response to hypoxia.

In the orthotopic model, sh-CD73 cells had a significantly lower tumor take, as compared with the sh-NT cells. Although we did not address it here, this finding may be related to tumor immunity, as cells with high CD73 expression can evade anti-tumor immunity in immunocompetent mice that were used in these studies. Furthermore, in line with decreased growth and migration in vitro, cells with suppressed CD73 expression formed significantly smaller tumors and less lung metastases than control cells^13,15,17^. Similar effects on lung metastases have also been described by other investigators^16,40^. Interestingly, we did not detect a significant difference in liver metastases, suggesting that CD73 may mediate specifically lung metastases. This notion requires, however, further investigation. Finally, the decreased metastasis by sh-CD73 cells may be associated with EMT, as suggested by the differences in E-cadherin and vimentin expression between sh-CD73 and sh-NT cells in vitro and in vivo. This observation was further confirmed at mRNA level, using a clinical cohort of breast tumors. Furthermore, in agreement with our results, vimentin-positive EMT was associated with more aggressive TNBC tumors^41^.

One caveat of the in vitro study was that APCP is a competitive inhibitor. Its efficiency and metabolic stability are lower when the concentration of substrate is high compared to allosteric inhibitors^42^. However, we demonstrated that blocking of CD73 by APCP had significant effect on cellular behavior and EMT progression in TNBC cells. Interestingly, CD73 deficiency did not regulate EMT markers in human mammary epithelial cells^27^. Recent studies have shown that adenosine-regulated G-coupled adenosine receptors A_2B_ or A_3_ induced EMT progression^43,44^. Except adenosine, purinergic ATP receptors mediated E-cadherin expression and other EMT-related markers in cancer cells^45,46^. These finding might point to other regulations, beyond CD73-derived adenosine^47^. Further studies are required to investigate CD73 catalytic activity with more potential selective inhibitors^48–50^.

CD73 is an emerging target for cancer therapy, the main rationale being inhibition of CD73 associated immunosuppression^7,8,51^. However, our data further suggests that anti-CD73 therapy could suppress progression and metastases of cancer cells also independent of the immune effects, possibly through inhibition of EMT^11,52^. It is likely, that there are also other mechanisms involved. For example, lack of CD73 was also suggested to prevent tumor growth, through inhibition of angiogenesis^53^.

In conclusion, our current data and previous publications suggest that CD73 effects on TNBC progression are likely mediated by both tumor cell intrinsic behavior, as further demonstrated here and also by immunology, as previously demonstrated by other groups. These data further support targeting CD73 in the treatment of TNBC.

## Materials and methods

### Cell culture

Human MDA-MB-231 and mouse 4T1 TNBC cells (ATCC, Manassas, VA, USA) were cultured in complete Dulbecco’s modified Eagle’s medium supplemented with 10% heat-inactivated fetal bovine serum (Gibco, EU Approved, South American Origin), 1% L-glutamine, 1% MEM NEAA, and 1% penicillin–streptomycin (all from Gibco, Life Technologies, Paisley, UK). The cells were cultured in 1% or 5% O_2_, or normoxia (21% O_2_). For hypoxia experiments, the cells were seeded and cultured in normoxia for 24 h, prior to placing them in hypoxia (InvivO_2,_ Ruskin Technology Ltd.).

### RNA interference

CD73 was downregulated in the 4T1 cells through stable small hairpin RNA (shRNA) transduction, using mouse-specific lentiviral particles, according to manufacturer’s recommendations (Mission lentiviral transduction particles, Sigma-Aldrich). Non-targeting particles (NT-shRNA) were used as a control. Stably transfected cells were selected using 4 µg/ml puromycin (Gibco, Life Technologies, UK) in complete culture medium.

### Quantitative real-time PCR

Total RNA was isolated with RNeasy RNA isolation kit according to manufacturer’s instructions (Qiagen). Purified RNA (1 μg) was converted to cDNA using Oligo-d(T) 18 mRNA primers (New England BioLabs) with Maxima RT enzyme, dNTP and RNAse inhibitor (all from Thermo Fisher Scientific). Q-PCR was performed using SYBR Green qPCR kit (Bio-Rad). CD73 primers were purchased from Bio-Rad Finland Oy. Primer sequences for TBP and SP1 primers are presented in Supplementary Table 1. The results were analyzed using the delta-delta Ct-method by first adjusting the Ct-values to that of the housekeeping gene TBP.

### Western blotting

Cells were cultured in complete culture medium and harvested after 24 h exposure to hypoxia in RIPA buffer (Thermo Fisher Scientific). Protein amounts were measured using bicinchoninic acid (BCA) protein assay (Thermo Fisher Scientific). The membranes were incubated with 5′-Nucleotidase/CD73, E-cadherin, vimentin HIF-1α, β-actin and α-tubulin primary antibodies overnight at 4 °C (Supplementary Table 2). Secondary detection was performed with anti-rabbit 800CW and anti-mouse 680CW antibodies (1:2000, IRDye, LI-COR). The emitted fluorescence was detected with Li-Cor Odyssey CLx imaging system.

### Immunofluorescence

Cells were seeded at the density of 1 × 10^4^ on sterile coverslips to attach overnight. After 24 h, cells were fixed with 4% paraformaldehyde in PBS for 15 min. The coverslips were incubated with anti-CD73 antibody (1:200, Novus, NBP2-158015) overnight at 4 °C. Secondary anti-rabbit AlexaFluor594 and phalloidin Fluor488 (both Invitrogen) labelled antibodies were applied for 1 h at RT. DAPI was used as a nuclear counterstain. Cell protrusions were manually measured using ImageJ/Fiji 1.52p^54^. All images were captured with Nikon Ti2-E fluorescence microscope.

### Thin layer chromatographic (TLC) analysis of CD73 activity

Cells were seeded overnight onto 96-well flat bottom clear plates at a density of 5 × 10^3^ cells/well. CD73 activity was determined by incubating the cells for 30 min at 37 °C in a final volume of 100 μl RPMI-1640 medium containing 5 mM β-glycerophosphate and 400 μM AMP with tracer [2, 8-^3^H] AMP (American Radiolabeled Chemicals Inc., Campro Scientific, The Netherlands). Aliquots of the mixture (8 μl, ~ 5 × 10^4^ dpm/spot) were applied onto Alugram SIL G/UV254 sheets (Macherey–Nagel, Germany). [^3^H]AMP and its dephosphorylated metabolite [^3^H]adenosine were separated by TLC and quantified by scintillation β-counting, as described elsewhere^55^. For enzymatic CD73 inhibition, parental MDA-MB-231 and 4T1 cells were pre-treated for 30 min with 100 µM of Adenosine 5′-(α,β-methylene) diphosphate (APCP, Merck Life Science OY, Finland) prior to addition of [^3^H]AMP substrate.

### Cell migration assays

For migration assays, the cells were cultured for 24 h to confluence on ImageLock 96-well plates (Essen Bioscience) in normoxia and hypoxia. For experiments with parental MDA-MB-231 or 4T1 cells, 100 µM APCP was added to the culture medium. Scratch wounds were made with WoundMaker (Essen Bioscience), after which the wells were filled with fresh medium. Images were captured every 6 h for 24 h with the IncuCyte S3 imaging system (Essen Bioscience). Wound confluences were analyzed using IncuCyte 2018B software.

### Cell viability assays

Cell viability was measured using WST-8 Cell Proliferation Assay Kit (Dojindo, Biotop Oy, Denmark) after 24 h exposure to normoxia, 1%, or 5% hypoxia. Parental MDA-MB-231 and 4T1 cells were treated with vehicle or APCP. The levels of WST-formazan were quantified spectrophotometrically using Tecan ULTRA microplate reader (Tecan AG, Austria) at 450 nm.

### Proliferation assays

For proliferation studies, 5 × 10^3^ 4T1 sh-NT and sh-CD73 cells were seeded on 96-well plates. Cell growth was assessed for 60 h, to allow the cells to reach confluence, after which the cell density was analyzed using IncuCyte 2018B software.

### Organotypic 3D cultures

Organotypic 3D cultures were prepared as described previously^38^. A growth matrix was prepared with a mixture of Matrigel (Corning) and collagen type I (Becton Dickinson) at a ratio of 8:2. Bottom wells of 96-well Angiogenesis µ-plates (Ibidi) were filled with 10 µl of 50% matrix diluted in medium and incubated at + 37 °C for 1 h. Cell suspensions were then mixed with 25% matrix diluted in medium and placed on top of the polymerized bottom gel. With this approach, one tumor organoid was generated from a single cell. The upper gels including the cells were allowed to polymerize at 37 °C for 3–4 h or overnight. The wells were then filled with complete culture medium and changed every 2nd–3rd day.

### Live cell staining and confocal image acquisition

The organoids were stained with Calcein AM (Invitrogen) to visualize living and dead cells. Confocal stacks were acquired with a Zeiss AxioVert 200 M microscope, equipped with Yokogawa CSU22 spinning disc unit using Plan-Neofluar 5× objective. Maximum intensity projections were created and background noise was reduced by normalization using the SlideBook software (Intelligent Imaging Innovations Inc.).

### Morphometric organoid analysis

Organoid projections were segmented using the AMIDA software^39^. The software segments multicellular structures and assigns values for selected cancer-relevant parameters. Raw numerical data were statistically processed and visualized using R (version 3.6.2) (http://cran.r-project.org) within the Rstudio (version 1.3.1073) interface^56,57^.

### Orthotopic mouse model

Four-week-old female Balb/c mice (Balb/cOlaHsd) were obtained from Envigo (the Netherlands). Animals were maintained in pathogen-free temperature- and humidity-controlled rooms under a 12 h light/dark cycle and provided standard supply of soya-free laboratory food and tap water ad libitum. The mice were inoculated with sh-NT and sh-CD73 4T1 cells (3 × 10^4^ cells in 100 μl PBS per mouse) orthotopically into the 4th mammary fat pads (n = 24/group). Starting 1 week after inoculation, tumor dimensions were measured and tumor volumes were counted as described^58^. Body weights were measured twice per week throughout the experiment. The animals were sacrificed 30 days after tumor inoculation by CO_2_ inhalation. Tumor and lung tissues were dissected for histology and immunohistochemistry.

### Histology and immunohistochemistry

Mouse tissue samples were prepared into paraffin blocks with standard methods. Hematoxylin and eosin (H&E) staining was performed to detect metastases. Metastases were analyzed blindly by two independent researchers. For immunohistochemistry (IHC), the slides were incubated with anti-CD73, anti-pHH3, anti-vimentin and anti-E-cadherin antibodies overnight at 4 °C. The slides were incubated with biotinylated secondary mouse or rabbit antibodies (BA-2000 Vector, UK) at room temperature for 1 h, followed by incubation with avidin/biotinylated enzyme complex (ABC) for 1 h, 3,3′-diaminobenzidine (DAB) for 15 min and counterstaining with hematoxylin for 10 s (both Vector laboratories, UK). The slides were scanned using Pannoramic 250 slide scanner (3DHISTECH Ltd, Hungary). IHC results were analyzed with QuPath-0.2.0_m4 software^59^. The results are shown as a percentage of DAB-positive cells of the total area. Necrotic areas and tissue folds were excluded manually and stromal cells were excluded by the size. The scripts for CD73, pHH3, vimentin and E-cadherin analysis are presented in Supplementary Table 3.

### Statistical analysis

The results are shown as mean ± SD of three independent experiments. All analyses were performed using GraphPad Prism version 7.0 (GraphPad Software Inc, San Diego, CA, USA). Data were analyzed for statistical significance using two-tailed Student’s t-test and one-way ANOVA. The differences for which p was < 0.05 are reported as statistically significant. Comparisons between CD73 and E-cadherin or vimentin mRNA expressions in human breast cancer specimens were based on cBioPortal METABRIC database in 1904 samples^29–31^.

### Ethical approval

All procedures performed in studies involving animals were cared for in accordance with the Project Authorization Board of Finland (license No ESAVI/7015/2020) in accordance with the 2010/EU/63 EU Directive on the protection of animals used for scientific purposes and the ARRIVE guidelines^60^. All data obtained from cBioPortal is publicly available and completely anonymized, therefore, further approval for its use was not required.

## Supplementary Information

Supplementary Information.

## Abbreviations

AMP: Adenosine monophosphate
APCP: Adenosine 5′-(α,β-methylene)diphosphate
ATP: Adenosine triphosphate
BCA: Bicinchoninic acid
Coll: Collagen
ECM: Extracellular matrix
EMT: Epithelial–mesenchymal transition
ER: Estrogen receptor
HER2: Human epidermal growth factor-2
IHC: Immunohistochemistry
Mtg: Matrigel
NOX: Normoxia
PBS: Phosphate buffered saline
pHH3: Phospho-histone H3
PR: Progesterone receptor
RT: Room temperature
SP1: Specificity protein 1
TBP: TATA-box binding protein
TNBC: Triple-negative breast cancer
