# Supplementary material for: CD73 facilitates EMT progression and promotes lung metastases in triple-negative breast cancer
Source: Sci Rep. 2021 Mar 16;11:6035. doi: 10.1038/s41598-021-85379-z (PMC7966763; doi:10.1038/s41598-021-85379-z)
Supplement: Supplementary file 1 — Supplementary Information. [file 41598_2021_85379_MOESM1_ESM.pdf]

Supplementary information

CD73 facilitates EMT progression and promotes lung metastases in triple-negative breast cancer

Nataliia Petruk[1,2], Sanni Tuominen[1,2,3], Malin Åkerfelt[1,2,4], Jesse Mattsson[1,2], Jouko Sandholm[5], Matthias Nees[1,2,6], Gennady G Yegutkin[7], Arja Jukkola[8], Johanna Tuomela[†], Katri S. Selander[9*]

[1]Institute of Biomedicine, University of Turku, Turku, Finland

[2]Western Cancer Centre FICAN West, Turku, Finland

[3]Preclinical Imaging Laboratory, Turku PET Centre, University of Turku

[4] Faculty of Science and Engineering, Cell Biology, Åbo Akademi University, Turku, Finland

[5]Turku Bioscience Centre, University of Turku and Åbo Akademi University, Turku, Finland

[6]Department of Biochemistry and Molecular Biology, Medical University in Lublin

[7]MediCity Research Laboratory, University of Turku, Turku, Finland

[8]Department of Oncology, Tampere University Hospital, Tays Cancer Center, Tampere, Finland

[9]Department of Oncology, Oulu University Hospital, Oulu, Finland

[†]Deceased author

*Corresponding author: Dr. Katri S. Selander, M.D., Ph.D

e-mail: Katri.Selander@ppshp.fi

Supplementary Figure S1

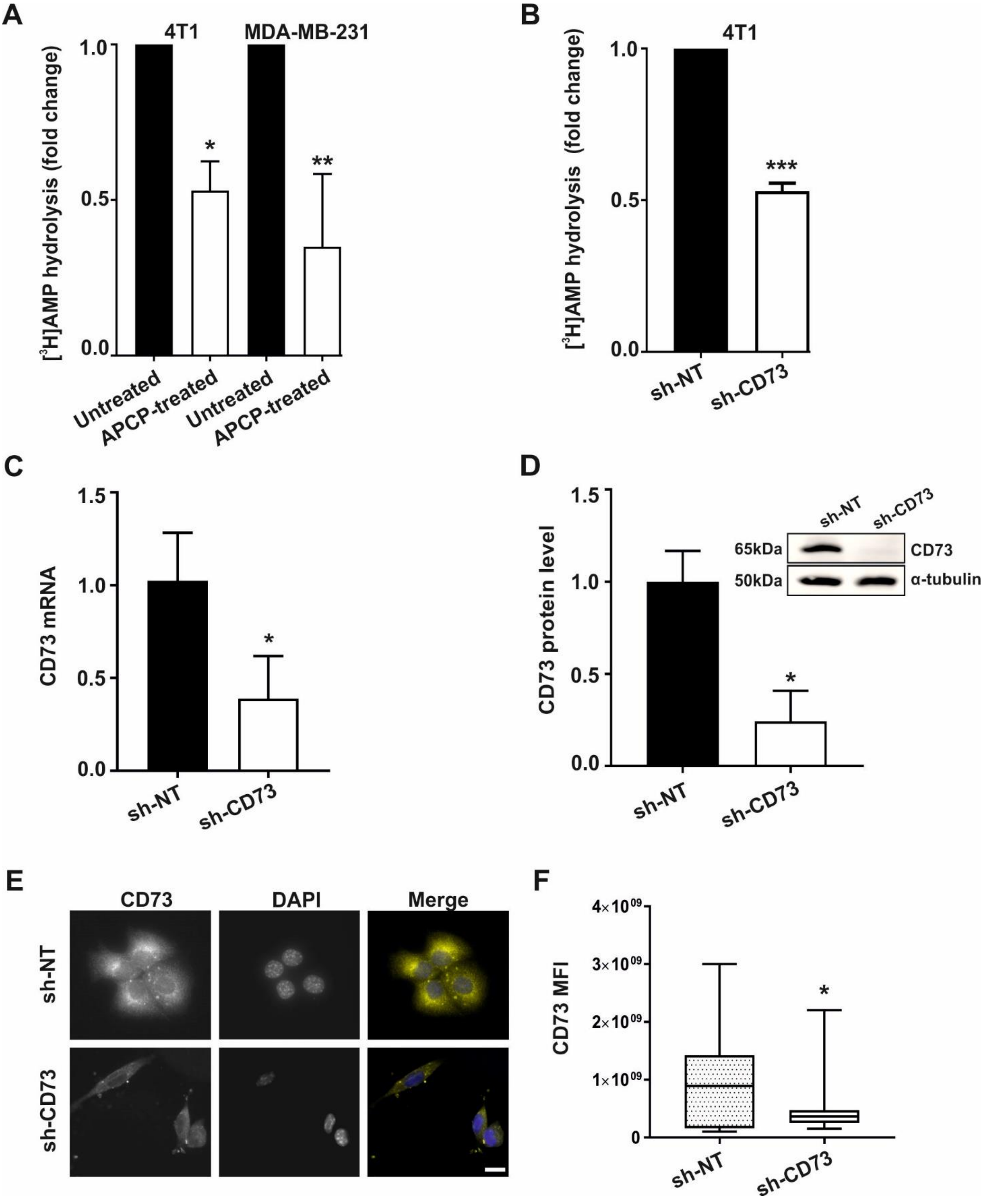

Figure S1. Downregulation of CD73 expression in TNBC cells. CD73 activity was determined by TLC as the rate of [^{3}H]AMP hydrolysis to [^{3}H]adenosine in (**A**) 4T1, MDA vehicle and APCP-treated cells, or (**B**) 4T1 sh-NT and sh-CD73 cells. The results are expressed as a fold change of [3H]AMP-hydrolyzing activity determined in untreated cells or in sh-NT cells (mean ± SD, n=3). (**C**) CD73 mRNA expression was analyzed by qPCR. The bars represent CD73 mRNA relative to the housekeeping gene TBP, data is expressed as mean ± SD, n = 3. (**D**) CD73 protein expression in 4T1 shRNA cells. Data is expressed as CD73 protein level relative to α-tubulin, the bars represent mean ± SD, n =3.

(**E**) Representative pictures and (**F**) intensity levels of CD73 in sh-NT and sh-CD73 4T1 cells. CD73 expression was determined by immunostaining. The data is expressed as mean fluorescence intensity (MFI) from 3 independent experiments, the bars represent mean ± SD, n = 25 cells. * $P<0.05$ and *** $P<0.001$ are considered to be statistically significant compared to representative controls, by two-tailed Student's *t* – test.

Supplementary Figure S2.

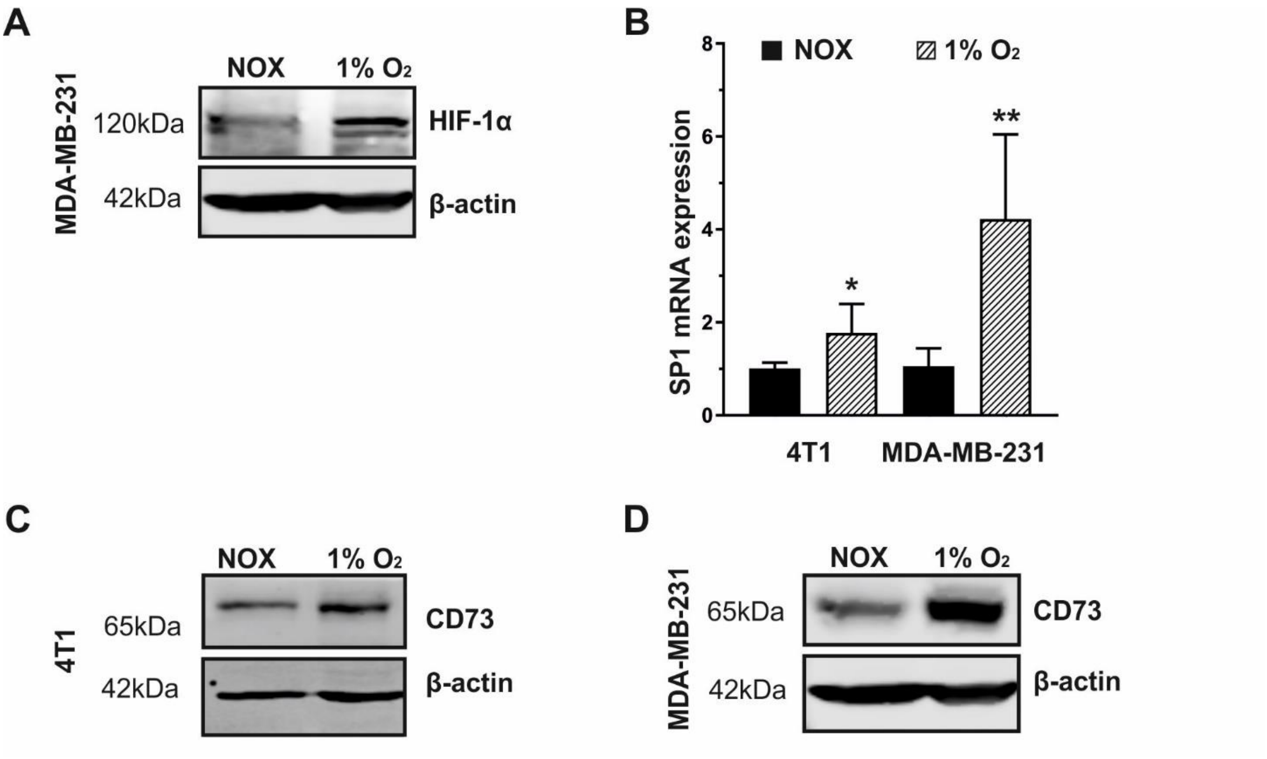

Figure S2. (**A**) HIF1α protein expression in MDA-MB-231 in normoxia (NOX) and after 24 h of hypoxia (1% O_2). (**B**) SP1 mRNA expression in 4T1 and MDA-MB-231 cells exposed to 1% O_2. The bars represent mean ± SD, n = 3. (**C**) CD73 protein expression in 4T1 and (**D**) MDA-MB-231 cells in normoxia and hypoxia (24h). * $P < 0.05$ and ** $P < 0.01$ were considered to be statistically significant compared to representative control cells, by a two-tailed Student's *t* – test.

Supplementary Figure S3

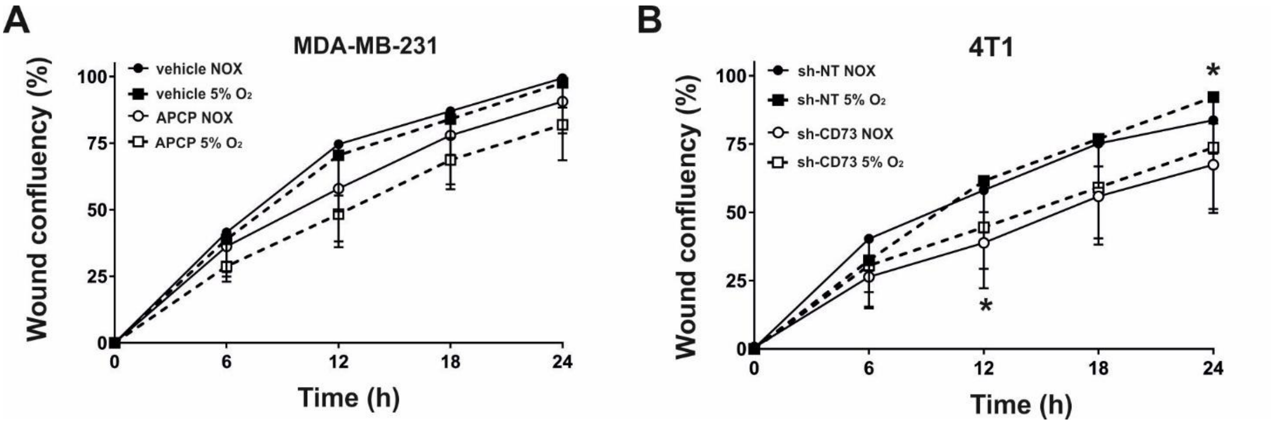

Figure S3. Cancer cell migration in 5 % O_2. Scratch wound assay with (**A**) APCP-treated MDA-MB-231 cells and (**B**) 4T1 sh-NT and sh-CD73 in normoxia and 5% O_2. Wound confluences were analyzed using IncuCyte 2018B software (Essen Bioscience). The data is expressed as mean ± SD, n=3. * $P < 0.05$ vs representative controls in normoxia, by one-way ANOVA with a Dunnett post-test.

Supplementary Figure S4

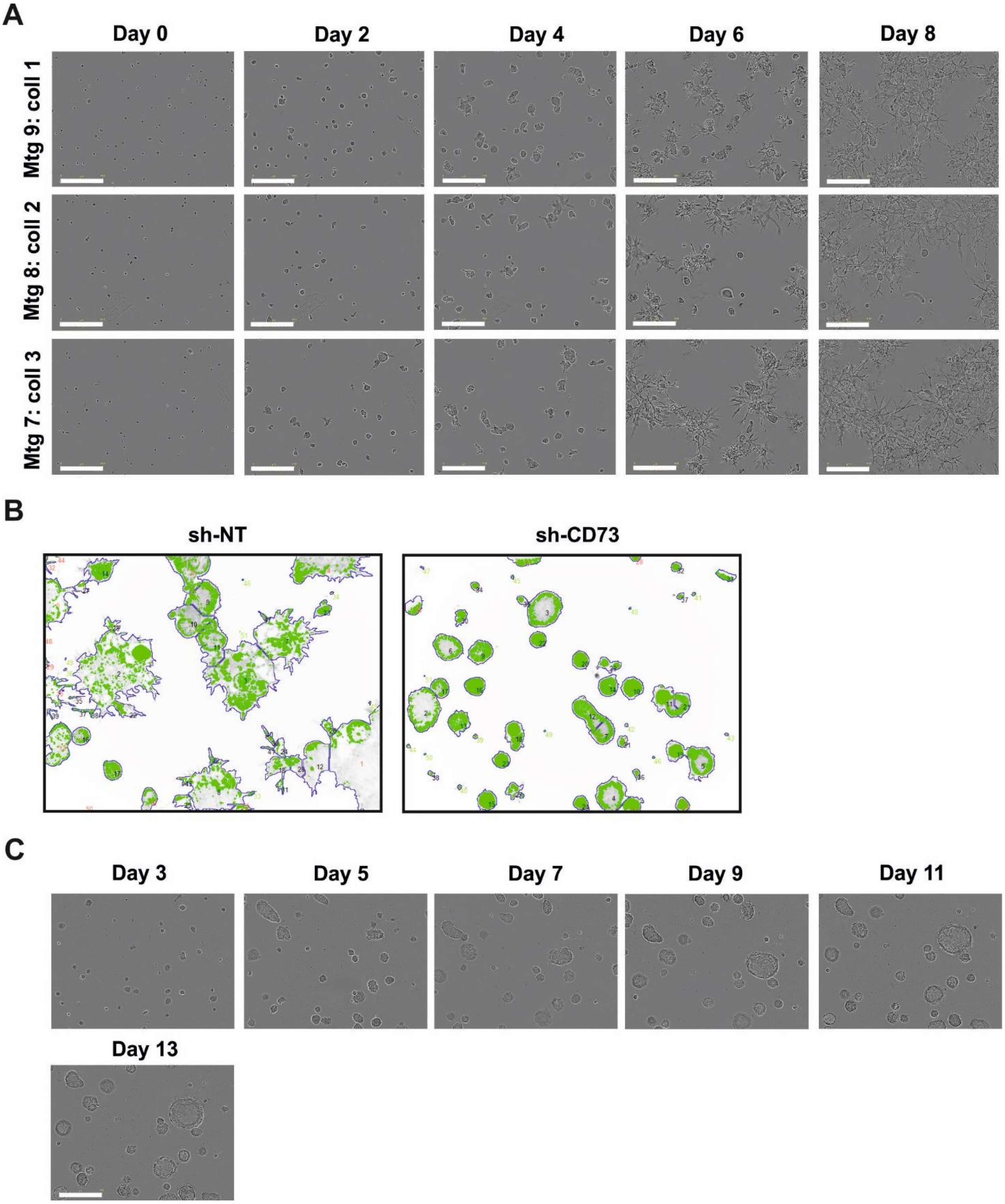

Figure S4. Optimization and analysis of 4T1 organotypic 3D cultures. (**A**) Different ratios of matrix components Matrigel (Mtg) and collagen type 1 (coll) were tested to provide a suitable environment for 4T1 cells that effectively promotes both polarization of organoids, and emergence of invasive structures. Mtg:coll ratio of 8:2 was selected for further studies. Scale bar: 400 µm. (**B**) Representative segmentation of live-cell spinning disk confocal images of 4T1 sh-NT and sh-CD73 growth for 6 days in 3D culture. (**C**) sh-CD73 cells grown in 3D culture. Representative images from days 3 to 13 are shown. Scale bar: 400 µm. Organoids were segmented and analyzed with AMIDA software.

Supplementary Figure S5

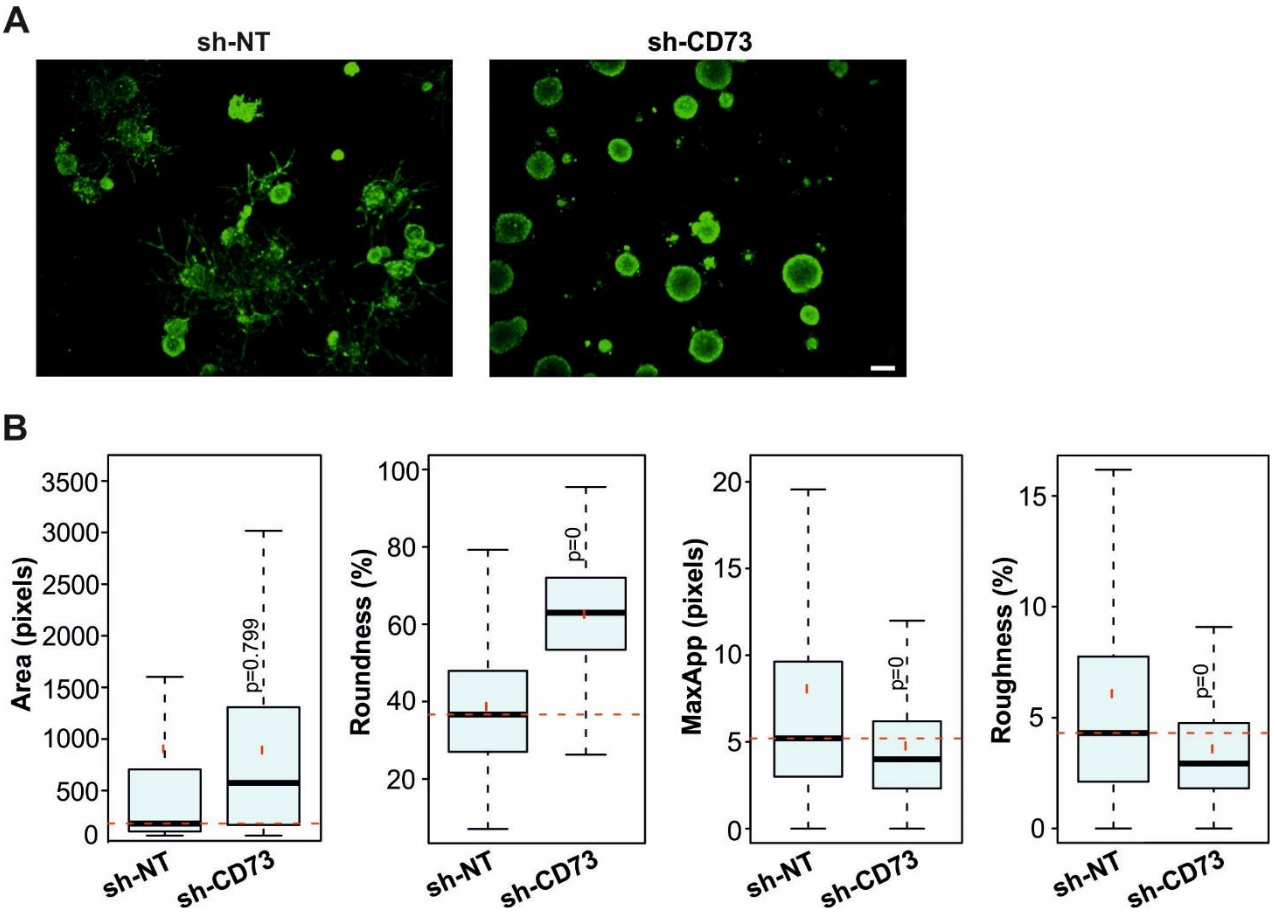

Figure S5. CD73 suppression prevents TNBC organoid invasion in hypoxia. (**A**) Confocal spinning disk microscopy images of sh-NT and sh-CD73 3D culture after 6 days under hypoxia. Calcein AM was used to detect living cells (green). Scale bar: 100 µm. (**B**) Box and whisker plots of selected parameters. Spheroid growth is indicated by the Area of the organoids. Roundness serves as a measure for the loss of the round organoid phenotype, and is associated with invasive properties. Roughness of the segmented structures and the maximal length of cellular protrusions emerging from the core structure (MaxApp) are quantitative measures for local invasion and cell motility. P=0 represented P < 0.001; sh-CD73 vs sh-NT spheroid formation.

Supplementary Figure S6

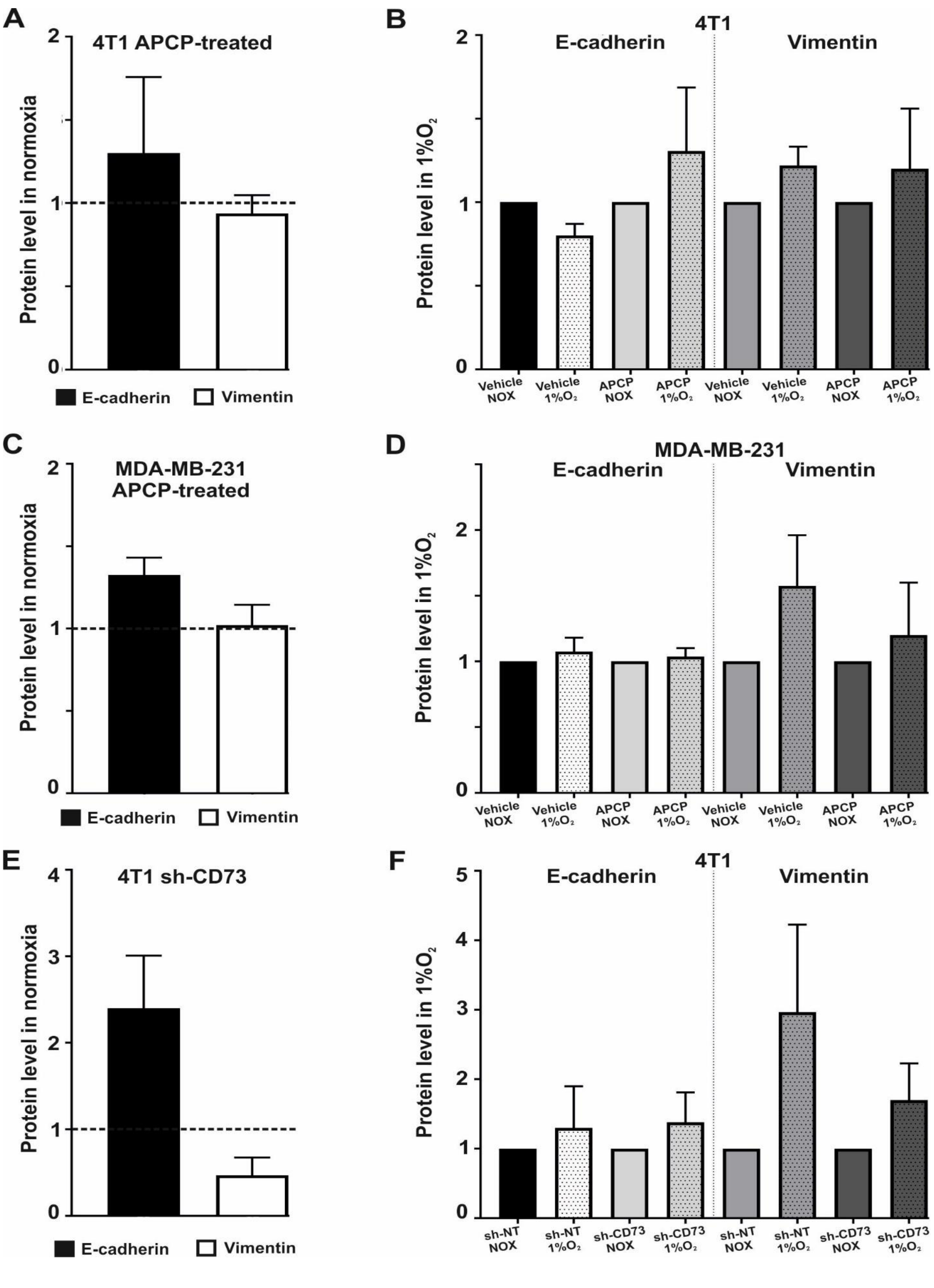

Figure S6. Quantification of E-cadherin and vimentin expression in normoxia and 1 % O_2. E-cadherin and vimentin protein expressions in 4T1 vehicle and APCP-treated cells in normoxia (**A**) and in 1 % O_2 (**B**). E-cadherin and

vimentin protein expressions in MDA-MB-231 vehicle and APCP-treated cells in normoxia (**C**) and in 1 % O_2 (**D**). E-cadherin and vimentin protein expressions in 4T1 sh-NT and sh-CD73 cells in normoxia (**E**) and in 1 % O_2 (**F**). The bars in A, C, and E represent fold-change in APCP-treated or 4T1 sh-CD73 cells as compared with untreated or 4T1 sh-NT cells in normoxia, respectively (set to 1 and indicated with the dotted line). The data is expressed as mean ± SEM, n=3. Statistical significance was analyzed by Wilcoxon rank test with theoretical median 1.

Supplementary Figure S7

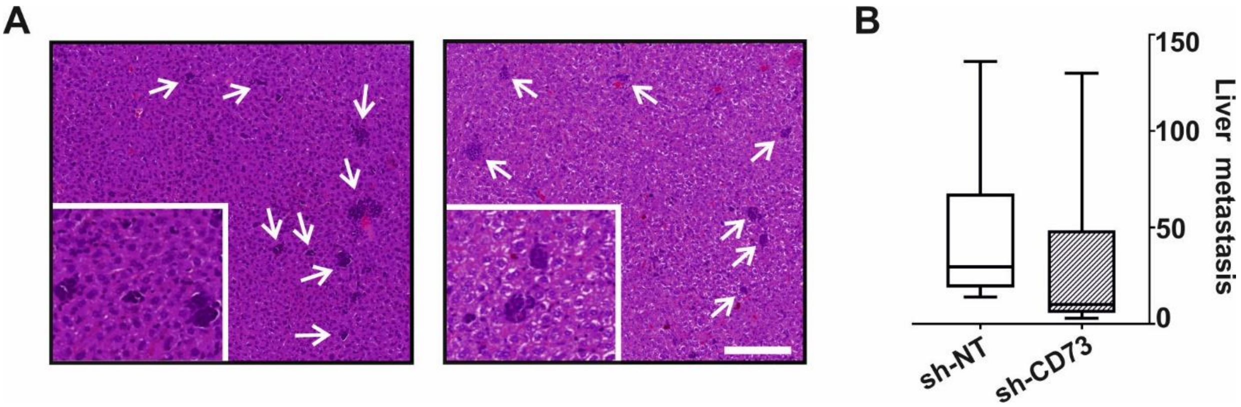

Figure S7. Number of liver metastasis in sh-NT and sh-CD73 tumors. (**A**) Representative H & E stainings from the liver of tumor bearing mice, metastases are indicated with white arrows. Scale bar 200 µm. (**B**) Numbers of liver metastases from sh-NT and sh-CD73 groups. Data is expressed as mean ± SEM.

Supplementary Figure S8.

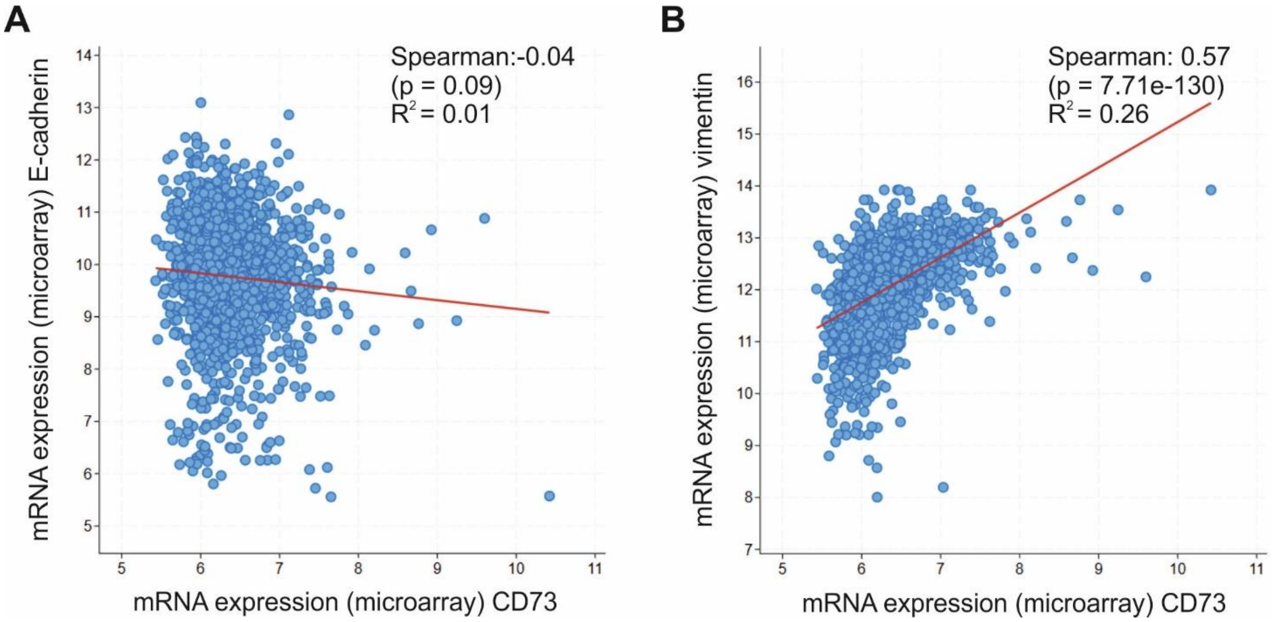

Figure S8. E-cadherin and vimentin in human breast cancer samples from METABRIC database. (**A**) The correlation of E-cadherin and CD73 in 1904 human breast cancer samples. (**B**) The correlation of vimentin and CD73 in 1904 human breast cancer samples.

Supplementary Figure S9. The original uncropped blots presented in Figures and Supplementary Figures.

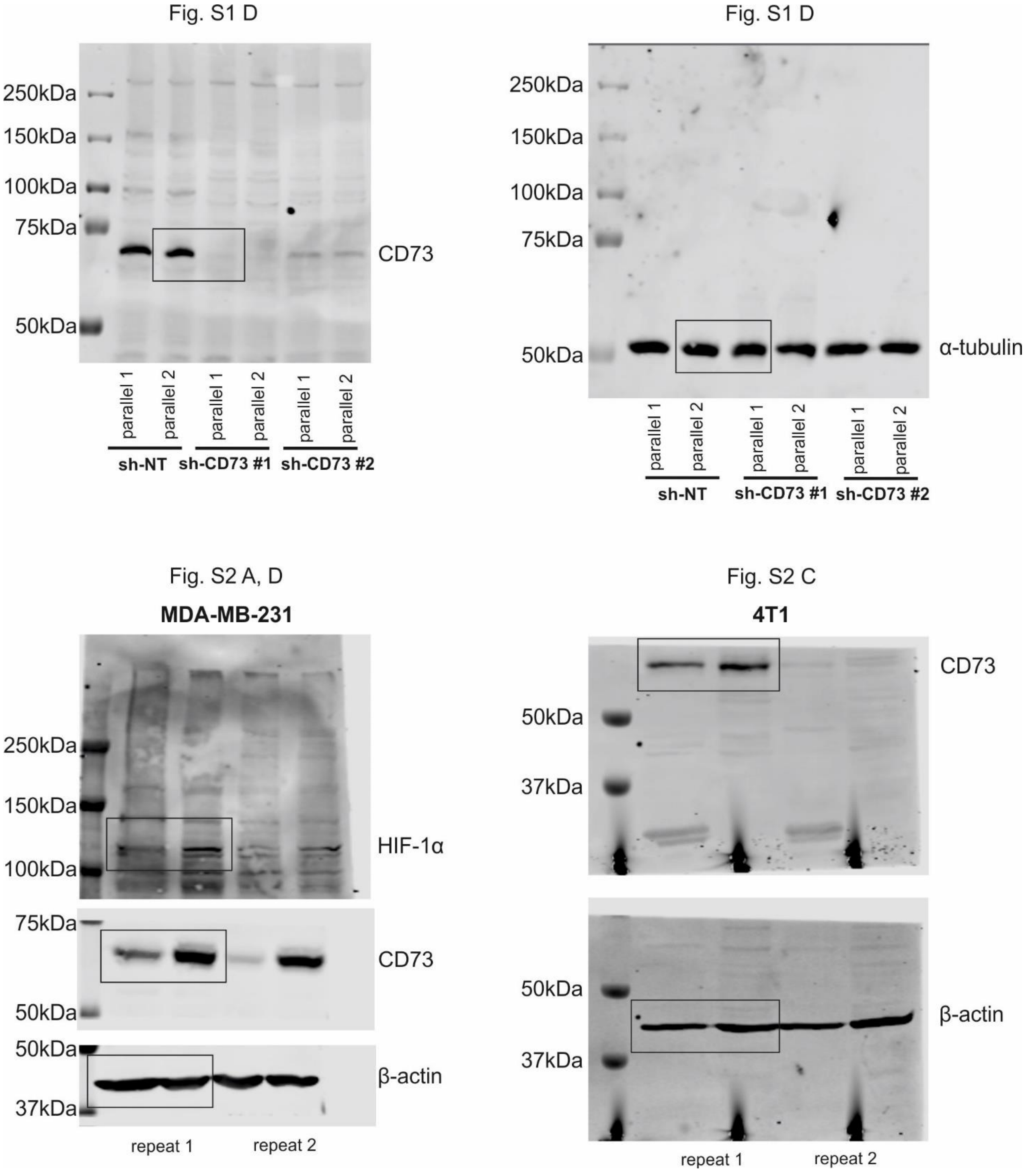

Supplementary Figure S10. The original uncropped blots presented in Figures and Supplementary Figures.

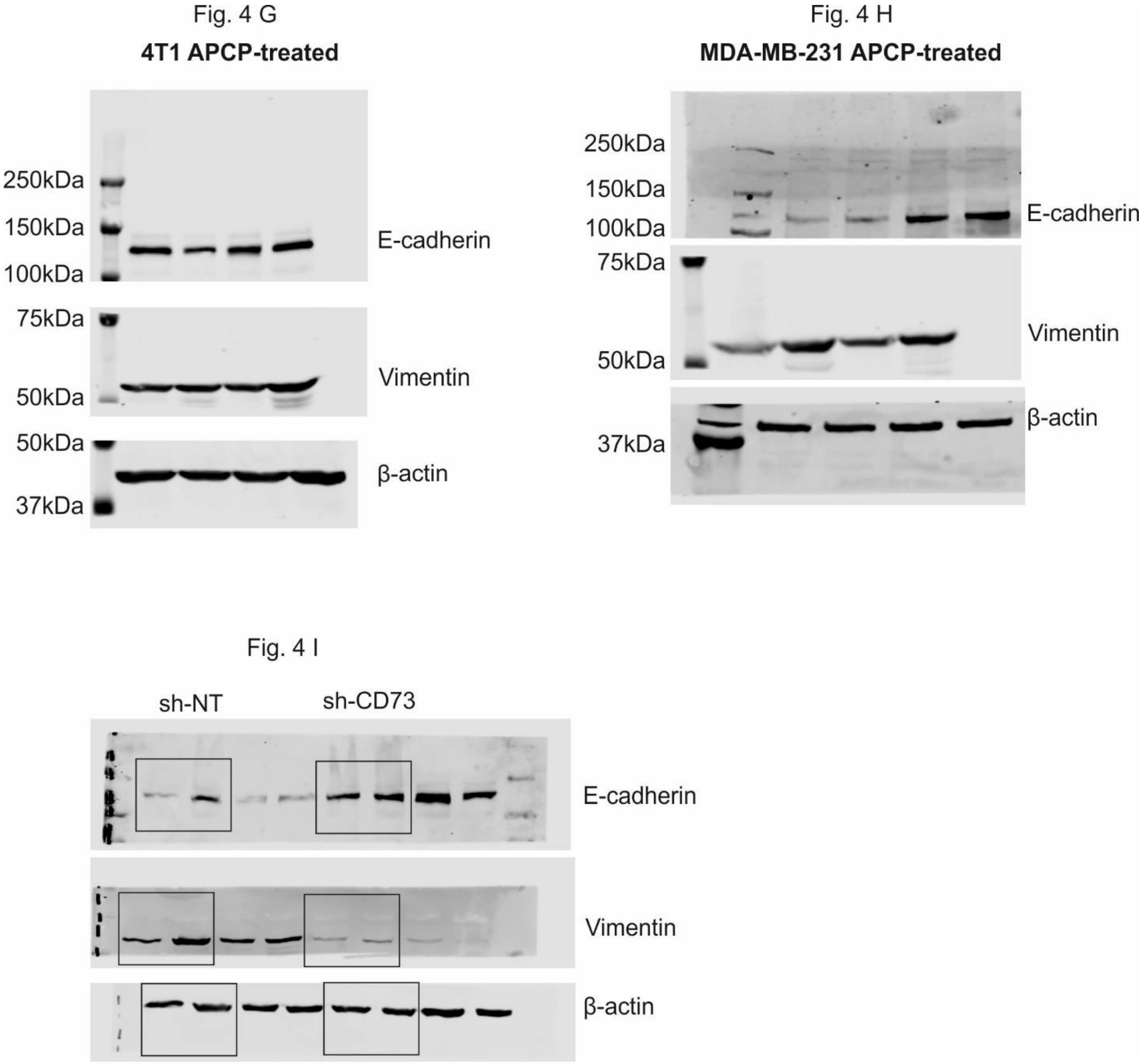

Supplementary table 1

Primers for quantitative polymerase chain reaction

Gene	Forward 5'– 3'	Reverse 5'– 3'
mTBP	GCA GCC TCA GTA CAG CAA TC	CTG CGG TAC AAT TCC AGA GC
hTBP	ACT TCA CAT CAC AGC TCC CC	GAA TAT AAT CCC AAG CGG TTT G
mSP1	GCA AGA CCT CAC ATC TCC GA	TTT CCC TTG GGT CTT ACT CAC C
hSP1	AGG CGA GAG GCC ATT TAT GT	TTC TCT CCC ATA GGC TCT TGC

Annealing temperature of 60°C for a total of 40 cycles

Supplementary table 2

List of primary antibodies

Primary antibody	Wester blotting dilution	IHC dilution	Manufacturer
5’-Nucleotidase/CD73	1:1000	1:200	NBP2-158015, Novus, UK
E-cadherin	1:1000	1:100	24E10, CST, USA
Vimentin	1:1000	1:200	D21H3, CST, USA
HIF-1α	1:500		610958, BD Bioscience, USA
β-actin	1:10000		A1978, Sigma-Aldrich
α- tubulin	1:20000		ab4074, Sigma-Aldrich
pHH3		1:500	9701, CST, USA

Supplementary table 3

QuPath scripts on analysis of immunohistochemical stainings from Figure 5

CD73 script

```
setImageType('BRIGHTFIELD_H_DAB');
setColorDeconvolutionStains('{"Name" : "H-DAB default", "Stain 1" : "Hematoxylin", "Values 1" : "0.76349 0.58762 0.26793 ", "Stain 2" : "DAB", "Values 2" : "0.12834 0.44649 0.88554 ", "Background" : " 255 255 255 "}');
runPlugin('qupath.imagej.detect.cells.PositiveCellDetection', '{"detectionImageBrightfield": "Hematoxylin OD", "requestedPixelSizeMicrons": 0.4,  "backgroundRadiusMicrons": 8.0,  "medianRadiusMicrons": 0.0, "sigmaMicrons": 1.5,  "minAreaMicrons": 20.0,  "maxAreaMicrons": 400.0,  "threshold": 0.1,  "maxBackground": 2.0,  "watershedPostProcess": true,  "excludeDAB": false,  "cellExpansionMicrons": 4.0,  "includeNuclei": true, "smoothBoundaries": true,  "makeMeasurements": true,  "thresholdCompartment": "Cytoplasm: DAB OD mean", "thresholdPositive1": 0.16,  "thresholdPositive2": 0.4,  "thresholdPositive3": 0.6000000000000001, "singleThreshold": true}');
```

pHH3 script

```
setImageType('BRIGHTFIELD_H_DAB');
setColorDeconvolutionStains('{"Name" : "H-DAB default", "Stain 1" : "Hematoxylin", "Values 1" : "0.8124 0.52465 0.25446 ", "Stain 2" : "DAB", "Values 2" : "0.32335 0.54612 0.77279 ", "Background" : " 255 255 255 "}');
runPlugin('qupath.imagej.detect.cells.PositiveCellDetection', '{"detectionImageBrightfield": "Optical density sum", "requestedPixelSizeMicrons": 0.5,  "backgroundRadiusMicrons": 8.0,  "medianRadiusMicrons": 0.0, "sigmaMicrons": 1.5,  "minAreaMicrons": 10.0,  "maxAreaMicrons": 400.0,  "threshold": 0.1,  "maxBackground": 2.0,  "watershedPostProcess": true,  "excludeDAB": false,  "cellExpansionMicrons": 5.0,  "includeNuclei": true, "smoothBoundaries": true,  "makeMeasurements": true,  "thresholdCompartment": "Cell: DAB OD mean", "thresholdPositive1": 0.13,  "thresholdPositive2": 0.4,  "thresholdPositive3": 0.6000000000000001, "singleThreshold": true}');
```

Supplementary table 4

QuPath scripts on analysis of immunohistochemical stainings from Figure 6

Vimentin script

```
setImageType('BRIGHTFIELD_H_DAB');
setColorDeconvolutionStains('{"Name" : "H-DAB default", "Stain 1" : "Hematoxylin", "Values 1" : "0.7048 0.66437 0.24874 ", "Stain 2" : "DAB", "Values 2" : "0.18976 0.4238 0.88566 ", "Background" : " 255 255 255 "}');
runPlugin('qupath.imagej.detect.cells.PositiveCellDetection', '{"detectionImageBrightfield": "Optical density sum", "requestedPixelSizeMicrons": 0.6,  "backgroundRadiusMicrons": 8.0,  "medianRadiusMicrons": 0.0, "sigmaMicrons": 1.5,  "minAreaMicrons": 30.0,  "maxAreaMicrons": 400.0,  "threshold": 0.1,  "maxBackground": 2.0,  "watershedPostProcess": true,  "excludeDAB": true,  "cellExpansionMicrons": 4.0,  "includeNuclei": false, "smoothBoundaries": false,  "makeMeasurements": true,  "thresholdCompartment": "Cell: DAB OD mean", "thresholdPositive1": 0.18,  "thresholdPositive2": 0.4,  "thresholdPositive3": 0.5,  "singleThreshold": true}');
```

E-cadherin script

```
setImageType('BRIGHTFIELD_H_DAB');
setColorDeconvolutionStains('{"Name" : "H-DAB default", "Stain 1" : "Hematoxylin", "Values 1" : "0.71407 0.62139 0.32247 ", "Stain 2" : "DAB", "Values 2" : "0.27092 0.53562 0.79982 ", "Background" : " 252 252 253 "}');
runPlugin('qupath.imagej.detect.cells.PositiveCellDetection', '{"detectionImageBrightfield": "Hematoxylin OD", "requestedPixelSizeMicrons": 0.5,  "backgroundRadiusMicrons": 8.0,  "medianRadiusMicrons": 0.0, "sigmaMicrons": 1.5,  "minAreaMicrons": 20.0,  "maxAreaMicrons": 400.0,  "threshold": 0.1,  "maxBackground": 2.0,  "watershedPostProcess": true,  "excludeDAB": false,  "cellExpansionMicrons": 4.0,  "includeNuclei": false, "smoothBoundaries": true,  "makeMeasurements": true,  "thresholdCompartment": "Cell: DAB OD mean", "thresholdPositive1": 0.15,  "thresholdPositive2": 0.4,  "thresholdPositive3": 0.6000000000000001, "singleThreshold": true}');
```
